# Oral Administration of Silybin Protects Against MPTP-Induced Neurotoxicity by Reducing Pro-inflammatory Cytokines and Preserving BDNF Levels in Mice

**DOI:** 10.1007/s12035-023-03485-7

**Published:** 2023-07-22

**Authors:** Ricardo J. Ramírez-Carreto, Víctor J. Zaldívar-Machorro, Dafne J. Pérez-Ramírez, Blanca E. Rodríguez-López, Claudia Meza, Esperanza García, Abel Santamaría, Anahí Chavarría

**Affiliations:** 1 Unidad de Investigación en Medicina Experimental, Facultad de Medicina, Universidad Nacional Autónoma de México, 06726, Ciudad de México, México; 2 Facultad de Química, Universidad Nacional Autónoma de México, 04510 Ciudad de México, México; 3 Laboratorio de Neuroinmunología, Instituto Nacional de Neurología y Neurocirugía Manuel Velasco Suárez, S.S, Ciudad de México, 14269 México; 4 Facultad de Ciencias, Universidad Nacional Autónoma de México, S.S, Ciudad de México, 04510 México

**Keywords:** BDNF, Fractalkine, Neuroinflammation, Neuroprotection, Parkinson’s disease, Silybin

## Abstract

Parkinson’s disease (PD) is the second most frequent neurodegenerative disease associated with motor dysfunction secondary to the loss of dopaminergic neurons in the nigrostriatal axis. Actual therapy consists mainly of levodopa; however, its long-term use promotes secondary effects. Consequently, finding new therapeutic alternatives, such as neuroprotective molecules, is necessary. Among these alternatives is silybin (Sb), the major bioactive flavonolignan in silymarin. Both exert neuroprotective effects, preserving dopamine levels and dopaminergic neurons when administered in the 1-methyl-4-phenyl-1,2,3,6-tetrahydropyridine (MPTP) mouse PD model, being probably Sb the potential therapeutic molecule behind this effect. To elucidate the role of Sb in the PD model, we determined the dose-dependent conservation of striatal dopamine content following Sb oral administration. Then, we evaluated motor deficit tests using the best dopamine conservative dose of Sb and determined a cytokine-dependent inflammatory profile status, malondialdehyde as an oxidative stress product, and neurotrophic factors content in the MPTP-induced mouse PD model. Our results show that oral Sb at 100 mg/kg dose conserved about 60% dopamine levels. Also, Sb improved motor deficits, preserved neurotrophic factors content and mitochondrial function, reduced lipid peroxidation, diminished proinflammatory cytokines to basal levels, enhanced fractalkine production in the striatum and substantia nigra, and increased IL-10 and IL-4 levels in the substantia nigra in the MPTP mice. Thus, oral Sb may be a potential pharmacological PD treatment alternative.

## Introduction

Parkinson’s disease (PD) is the second most frequent neurodegenerative disease in industrialized countries, with a population prevalence of approximately 0.3% [1], ascending from 2.5 million people with PD in 1990 to about 6.1 million patients in 2016 [2], and is estimated to increase to 8.7–9.3 million people over the world in 2030 [3]. PD is characterized by neuronal loss in dopaminergic nuclei in the central nervous system (CNS), such as substantia nigra and corpus striatum, followed by a subsequent deficit of the neurotransmitter dopamine (DA) [4]. Some neurodegenerative mechanisms involved are mitochondrial dysfunction, aggregation of misfolded α-synuclein, altered proteolysis, inflammatory changes, aging, and oxidative/nitrosative stress, which includes enhanced lipid peroxidation and oxidation of other biomolecules [5]. Clinically, PD presents motor [6] and non-motor [7] symptoms due to DA deficit, decreasing life quality.

Pharmacological treatments for PD are currently headed by levodopa, a precursor amino acid to DA, which increases DA levels and improves motor activity when administered to PD patients [8]. Currently, levodopa is considered the most effective therapy [5]; nonetheless, its chronic administration generates treatment resistance and undesirable adverse effects like dyskinesia [9, 10]. Hence, alternatives to levodopa treatment are required, such as neuroprotective agents.

In mice, the experimental administration of the pro-neurotoxin 1-methyl-4-phenyl-1,2,3,6-tetrahydropyridine (MPTP) induces a selective dopaminergic neuronal loss in substantia nigra and striatum by biochemical and cellular changes similar to PD. Thus, the MPTP model is widely used to evaluate the neuroprotective effects of bioactive molecules [11, 12].

Silymarin is the ethanolic extract from *Silybum marianum* seeds [13] and is composed of several molecules, such as the flavonolignans silychristin, silydianin, and isosilybin, among other components. However, the main bioactive component characterized in silymarin is silybin (Sb), which has two stereoisomers, A and B, in a racemic mixture [14]. In recent years, silymarin has been reported as an effective neuroprotector in a PD model when administered intraperitoneally [15], possibly due to its antioxidant and anti-inflammatory properties [16–18]. However, intraperitoneal is not a commonly used administration route in humans, so it is necessary to evaluate Sb neuroprotective effects on clinically relevant administration routes, such as orally, which eases application and comfort in patients [19].

Silymarin can act as a potential neuroprotective agent [15], yet, its neuroprotective mechanisms in the PD context remain unknown. Also, Sb has been reported as a potential therapeutic molecule for preventing neuronal depletion in the MPTP mouse PD model [20, 21]. This work evaluated the neuroprotective effect of Sb administered orally in the MPTP mouse model and the effect of Sb on bradykinesia, gross and fine motor skills, equilibrium, and muscle strength using the pole, traction, and beam tests. Also, we determined Sb’s impact on the striatal DA content, brain-derived neurotrophic factor (BDNF), and insulin-like growth factor 1 (IGF-1) levels, lipid peroxidation, mitochondrial function, cytokine production such as interleukin (IL)-6, -10, -4, -1β, tumor necrosis factor α (TNFα), and fractalkine ligand levels in the PD’s murine model.

## Materials and Methods

### Animals and Bioethical Considerations

Eight to ten weeks-old male C57BL/6J mice (25–30 g) were maintained with food and water ad libitum under a 12:12 light-dark cycle, 40% relative humidity, and 20–22°C at room temperature. Animals were acclimatized for two weeks before the experiments. All animal groups were euthanized by pentobarbital overdose three or seven days after their respective last pro-neurotoxin administration, as required for experiments, following the AVMA Guidelines for the Euthanasia of Animals [22]. All experimental procedures were performed following the Mexican Official Law of Animal Protection NOM-062-ZOO-1999 [23] and the Guidelines for the Use of Animals in Neuroscience Research of the Society of Neuroscience [24]. Ethics, Research, and the Internal Committee for the Care and Use of Laboratory Animals (CICUAL) committees of Facultad de Medicina, UNAM, approved all experimental procedures (FM/DI/127/2018, and CICUAL 001-CIC-2019). We reduced the number of mice used and their suffering or pain as much as possible.

### Administration Scheme and Experimental Design

**Fig. 1:**
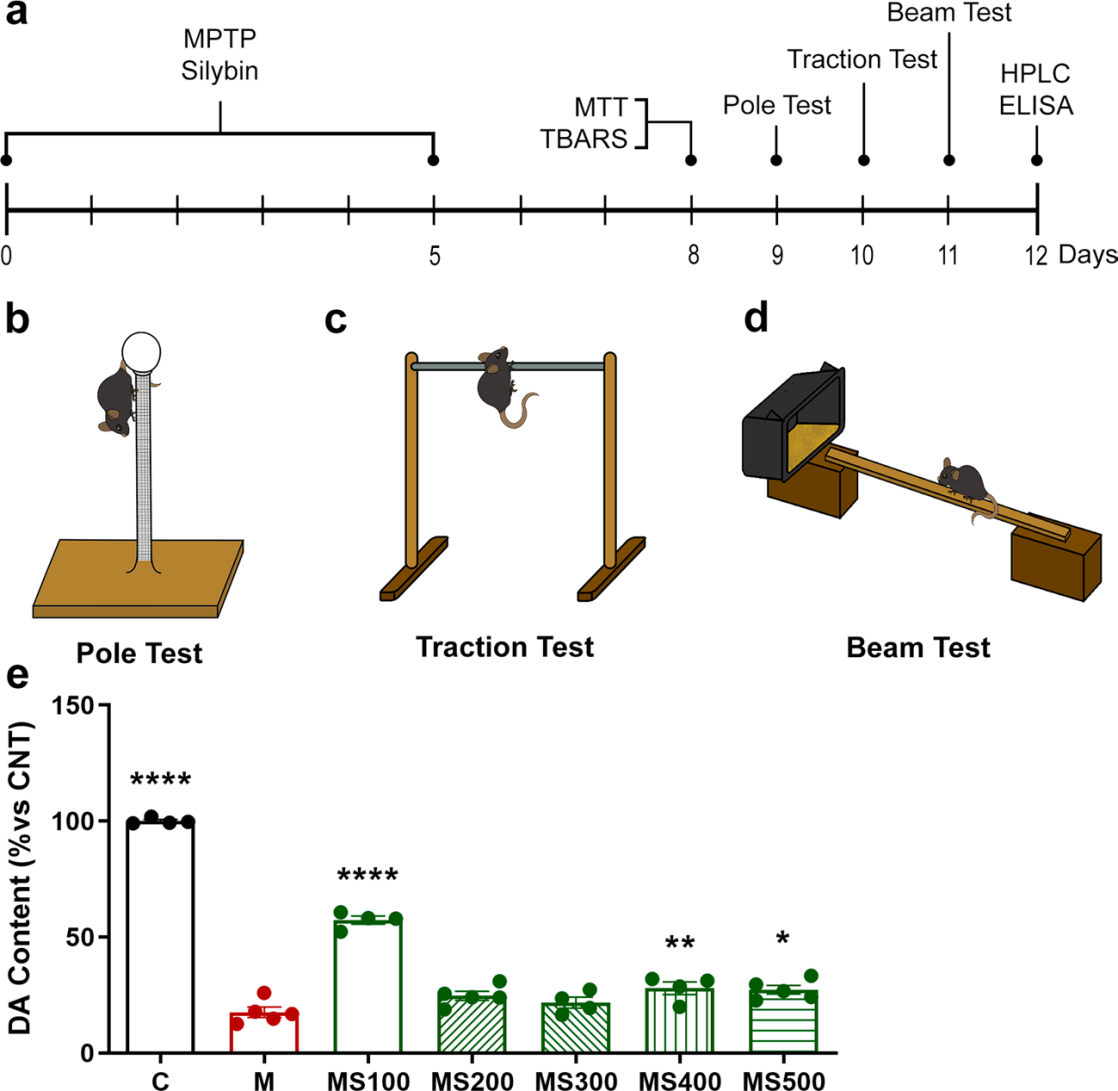
Silybin treatment attenuates MPTP-induced dopamine loss in the striatum. **a** C57BL6/J male mice were treated with intraperitoneal MPTP 30 mg/Kg doses daily followed by silybin (Sb) administration orally 30 min after each MPTP exposition for five consecutive days. Damage parameters assays were performed three days after the last MPTP administration; behavior tests were evaluated six days after the MPTP scheme; last HPLC and ELISA assays were performed seven days after the MPTP treatment. Motor behavior studies consist of three tests: **b** pole test, which evaluates bradykinesia and gross motor skills; **c** traction test to measure muscle strength, balance, and traction skills; and **d** beam test, which evaluates fine motor skills. **e** Striatal dopamine content was measured from mice administered with vehicles (C), MPTP (M), or MPTP coadministered with five different oral Sb dosages ranging from 100 to 500 mg/kg (MS100, MS200, MS300, MS400, and MS500). Data represent mean ± SEM (*n* = 4-5) and were analyzed by ANOVA, followed by Bonferroni post hoc test. **** *p* < 0.0001, ** *p* ≤ 0.01, and * *p* ≤ 0.05 compared to the M group

MPTP (Sigma-Aldrich, MO, USA) was administered intraperitoneally for five consecutive days at 30 mg/Kg to induce the Parkinsonian phenotype in mice, as reported previously [15, 25, 26]. The cumulative dose was 150 mg/kg to avoid necrotic cell death by MPTP doses [27]. MPTP was reconstituted in isotonic saline solution (ISS). For all Sb-treated experimental groups, 30 min after every daily neurotoxin administration, commercial standardized Sb (Sigma-Aldrich, MO, USA) was administered orally and dissolved in oleic acid (OA; Droguería Cosmopolita, CDMX, MEXICO) (Fig. 1a). Intragastric administration in mice was performed using an oral gavage, each mouse was trained for 3 days previous to the administration scheme for the restrain technique and recognition of the curved gavage needle (20 G × 1 ½ in). During the protocol performance, mice were gently restrained, immobilizing the head, but the procedure stopped if the animal vocalized or showed signs of distress. The total administration volume was calculated as 8 mL/kg. No restricted diet or fasting was needed.

Animals were divided into different groups: control group administered with the vehicles ISS intraperitoneally and OA orally (C), intraperitoneally MPTP-intoxicated mice orally administered with OA (M), 100 mg/Kg Sb-administered healthy mice with ISS intraperitoneally (S100), and MPTP-administered mice co-treated with Sb at 100 (MS100), 200 (MS200), 300 (MS300), 400 (MS400), or 500 (MS500) mg/Kg orally.

### Dopamine Determination

The analysis of striatal DA of all different experimental groups was performed by HPLC with electrochemical detection, as described by García et al. in 2008 [25]. Briefly, 7 days after the last MPTP administration dissected fresh striata were sonicated on ice with 300 μL of mixing solution (HClO_4_ 0.4 N + sodium metabisulfite 0.1%v/v), then centrifuged 4000 × *g*, 10 min at 4°C. Supernatants were collected, filtered, and stored at -70°C until use. Chromatographic analyses were performed with Perkin-Elmer LC-4C liquid chromatography with a BAS CC-5 electrochemical detector, and chromatograms were processed by the Perkin-Elmer Turbochrom Navigator 4.1 data station software. Alltech Adsorbosphere Catecholamine (100 × 4.6 mm) with 3 μm particles was used as the stationary phase, and the mobile phase was constituted by a phosphate buffer 0.1 M, pH 3.1 solution, with sodium octyl-sulfate 0.9 mM, EDTA 0.1 mM, and methanol 15%v/v. The flow rate was 1.4 mL/min. The potential was 0.8 V relative to an Ag/AgCl reference electrode. All samples were performed by duplicate and normalized by tissue weight. The results were expressed as a percentage of DA relative to control values (Fig. 1e).

### Behavioral Motor Tests

Three behavioral motor tests were performed on day nine (pole test), ten (traction test), and eleven (beam test) of the general experimental design, as shown in Fig. 1a, to confirm Sb’s impact on improving motor deficits induced by the MPTP treatment in mice.

### Pole Test

The pole test is useful to challenge bradykinesia [28, 29] and gross motor skills [30, 31]. The system and method were adapted from those employed by Sedelis [31] and Zhou [32], using an upper spherical base (2 cm in diameter) adhered to a 50-cm-long wood pole with 0.1 cm in diameter, non-adhesive gauze-wrapped to facilitate gripping, adjusted to a heavy wood square lower base (Fig. 1b). Each mouse started the test by looking down to the lower base, standing on the lateral side of the spherical base. Two parameters were documented: 1) time for turning down (T-Turn), which involves the time of the descending skill of each mouse; timing begins when the experimenter releases the animal and ends when one hind limb reaches the end of the pole [28, 32]. 2) The second parameter is the time landing (T-LA), which involves the same time beginning when the mouse is released but ends when the animal lands with its four limbs in the wood base [30, 33]. Both parameters were measured to compare their predictability. The test does not need previous training, and it was performed five times for each animal for 30 s as the time limit, and animals rested for 30 min between each test repetition. The test was performed on the third day after the last MPTP administration, and all intents were video recorded. Both parameters represent the meantime of their five repetitions and are reported as percentage time concerning control groups.

### Traction Test

The traction test measures mouse equilibrium behavior [34, 35] and muscle strength [28, 32], and evaluates fine motor skills when applied together with the challenging beam test. The traction test consists of placing the mouse on its forelimbs attached to a metal rope and challenging its capability to escape using its strength without falling. The employed system was adapted from Blanco’s rat version of the traction test [36] and followed Deacon’s protocol for its three bars mouse test [34] with some modifications. The apparatus consists of a 40-cm-long metal rope of 2 mm diameter, attached to two wooden poles (escape poles), which raise the rope about 50 cm in height. Also, a soft pillow base was placed below the rope at table level for animal protection in case of a fall (Fig. 1 c). This test does not need previous training and begins when the animal is placed in the middle of the rope in its forelimbs, time is documented for the animal to cross to any of the escape poles ending when the fist paw touches any of them and is reported as escape latency. For comparative purposes, we also reported the scoring method made by Deacon, assigned as one point for one to 5 s crossing, two points for 6 to 10 s crossing, three points for 11 to 20 s crossing, four points for 20 to 30 s, and five points for more than 30 s crossing. Events are not considered when falling and deserting during the cross latency. Desertions are non-move attaching limbs behavior or static standing on the metallic rope for grooming. 1) The escape ratio (ER), which is the index of tries that each mouse escaped satisfactorily and finished the test, detecting the deserting behavior, and is calculated as shown in equation 1. 2) The second parameter is the traction ratio (TR), which expresses the index of tries that each mouse did not fall off the system during the test, and is calculated as shown in Eq. 2. Both parameters were reported as the mean of repetitions. Each mouse was challenged to cross the rope five times in 30 s as the limit time and waited 30 minutes between each animal’s test repetition. The test was performed on the fourth day after the last MPTP administration, and all attempts were video recorded.

1 $$ER=\frac{Times\ crossed}{Total\ tries}$$

Equation 1. Escape Ratio (ER). ER describes the ratio of mice that satisfactorily finished the traction test in percentages compared to the control group as a deserting behavior parameter.

2 $$TR=\frac{Times\ tested\ without\ f\ all}{Total\ tries}$$

Equation 2. Traction Ratio (TR). TR describes the ratio of mice not falling during the traction test, expressed in percentages compared to the control group as a falling behavior parameter.

### Beam Test

The beam test is one of the most common behavioral tests for evaluating fine motor skills [33, 37] and balance behavior [30]. The system and method used were adapted from those established by Fleming [37] and Luong [38]. The test consists of crossing a flat bridge walking from the start point, which is 80 cm away from a black shaded homecage and ending in the last cm before arriving at the homecage. The apparatus used is composed of two acrylic bases that raise the system about 20 cm from the experimentation table with a 1-m-long, and 6-cm-width acrylic made flat beam on them, connecting both bases between them and a homecage provided with some food, bed, and a previously trained mouse for attracting challenged mice for crossing (Fig. 1d). This test requires three days of training based on Albutts’ results for erasing exploration behavior outside the system [39]. During the first two training days, mice were instructed to cross along the bridge, forcing them to cross and blocking any exploration or deserting behavior; on the last training day, mice were challenged to free-cross the bridge and watch if there were any distractions. A steel wire mesh is placed on the acrylic beam to make crossing more difficult on the test day. Each mouse was challenged to cross the bridge by 30 s as the limit time was up to five times with 30 min pause between each animal test repetition. The test was performed on the fifth day after the last MPTP administration, and all attempts were video recorded. Time crossing is quantified from the start to the endpoint and reported as the time of animal trials; i.e., crossing latency and individual mouse error counts were made, understood as errors as the limb slips and sudden stops. To improve predictability, we proposed calculating the relative cross error (RCE), dividing the error count by cross latency for erasing attrition bias as shown in Eq. 3, and calculating the number of mice that concludes the test satisfactorily using ER index. The attrition rate in the experimental groups was calculated identically as for the traction test in equation 3. Attrition is defined as unfinished mouse crossing by grooming, distraction, exploration, freezing, or change in walking direction.

3 $$RCE=\frac{Mouse\ mean\ error\ count}{Mouse\ cross\ latency}$$

Equation 3. Relative cross error (RCE). RCE describes the ratio of mouse errors during the time when crossing the challenging beam test as a fine motor skill parameter.

### MTT Reduction Assay

3-(4,5-dimethylthiazol-2-yl)-2,5-diphenyltetrazolium bromide (MTT) reduction assay was performed in homogenized brain tissue, using the methodology described by Pérez-De La Cruz et al. [40]. Three days after the last MPTP administration, fresh dissected striatal and nigral tissues were collected and processed in 100 μL of PBS 1× Solution (NaCl 137mM, KH_2_PO_4_ 1.5mM, Na_2_HPO_4_ 8.1 mM, and KCl 2.7 mM dissolved in deionized water) using sonication on ice and centrifugation as described on DA preservation curve. Samples were incubated with 10 μL of MTT 5 mg/mL for 60 min at 37°C and centrifuged at 15 300 × *g* for 3 min. Supernatants were discarded, and pellets were resuspended in 500 μL of acidic isopropanol solution (Isopropanol-HCL 96:4). Optical density readings were made by duplicates and detected at 570 nm using an EPOCH plate lector (BioTek, USA). Samples were normalized by tissue weight, and results were expressed as the percentage of MTT reduction relative to control groups.

### Lipid Peroxidation Assay

Thiobarbituric acid-reactive substances (TBARS) quantification assay was performed in homogenized mouse brain tissues using the methodology described by García et al. [25]. Three days after the last MPTP administration, fresh nigral and striatal dissected tissues were processed in 100 μL of PBS 1× Solution (NaCl 137mM, KH_2_PO_4_ 1.5mM, Na_2_HPO_4_ 8.1 mM, and KCl 2.7 mM dissolved in deionized water) using sonication on ice and centrifugation as described on DA preservation curve. Whole solution samples were added to 200 μL of TBA solution (0.375 g of thiobarbituric Acid + 15 g of trichloroacetic acid + 2.54 mL of HCl) and incubated at 94°C for 30 min in a hot water bath. A pinkish tone change is proportional to the concentration of the oxidized products. Samples were preserved on ice for up to 5 minutes and centrifuged at 3000 × g for 15 min. Supernatants were collected, and optical density was determined at 532 nm using an EPOCH plate lector (BioTek, USA). Samples were analyzed by duplicates and normalized by tissue weight. Results were expressed as the percentage of TBARS relative to control groups.

### Cytokines, Chemokines, and Neurotrophic Factors Detection by ELISA Titration

Levels of cytokines (TNFα, IL-1β, IL-4, IL-6, and IL-10), Fractalkine ligand, and neurotrophic factors (IGF-1 and BDNF) were determined in homogenized mice brain tissue using capture enzyme-linked immunosorbent assay (ELISA; DuoSet Mouse TNF-α DY410-05, DuoSet Rat IL-1β/IL-1F2 DY501-05, DuoSet Mouse IL-4 DY404-05, DuoSet Mouse IL-6 DY406-05, DuoSet Mouse IL-10 Dy417-05, DuoSet Mouse CX3CL1/Fractalkine DY472-15, DuoSet Mouse/Rat IGF-1/IGF-I DY791-15, and DuoSet Human/Mouse BDNF Dy248 ELISA kits, R&D Systems, Minneapolis, MN, USA), following the instructions of the manufacturer.

Three and 7 days after the last MPTP administration, dissected nigral and striatal tissues were collected and homogenized by sonication using 300 μL of lysis buffer solution (Tris 20mM, sucrose 0.25M, EDTA 2mM, EGTA 10mM, TritonX-100 1%) containing a protease inhibitor cocktail, then centrifuged at 4000 × *g* by 30 min at 4°C. Supernatants were collected and preserved at -40°C until use.

Samples were incubated on a capture antibody-coated plate for 24 h at 4°C with PBS-Tween 20 (0.05%)/0.5% BSA, washed three times, and incubated with a detection antibody for 2 h at room temperature. The attached antigen-antibody systems were detected using the TMB substrate (Sigma-Aldrich, MO, USA). Optical density readings were done at 450 nm and 570 nm. All assays were performed in duplicates; their sensitivities were 0.31 ng/ml for Fractalkine, 15.63 pg/mL for TNFα, IL-1β, and BDNF systems and 31.25 pg/mL for IL-4, IL-6, IL-10, and IGF-1 systems. Sample results were adjusted to pg/mg tissue using tissue weight and dilution factor.

### Statistical Analysis

Results from all experiments were subjected to normality (D’Agostino-Pearson test) and homoscedasticity (Brown-Forsythe test) tests to determine parametric data. Parametric data were analyzed using One-way ANOVA tests with Bonferroni (dopamine determination curve) or Tukey post hoc. Non-parametric tests were analyzed through Kruskal-Wallis tests using Dunn post hoc. Both statistical analysis profiles consider a *p* < 0.05 confidence level, and results were expressed as mean values ± SD or median with interquartile range (GraphPad Prism 9, La Jolla, CA).

## Results

### Orally Administered Sb Preserves Striatal DA Levels in MPTP-Intoxicated Mice

As reported previously, MPTP administration significantly depleted the striatal DA content (82.43% below the control group; *p* < 0.0001) [25]. Previously, we evaluated a dose-response curve for the whole *S. marianum* extract (silymarin), and we determined that 100 mg/Kg injected intraperitoneally had the best neuroprotective potential [15]. Due to silymarin and silybin's low bioavailability [41, 42], in this work, we evaluated the dose-response curve for orally administered Sb in higher doses than 100 mg/kg and showed that also the MS100 group preserved striatal DA levels (57.27%) compared to the M group (*p* < 0.0001) (Fig. 1e). The MS400 and MS500 groups preserved DA levels in less proportion (27.92% *p* = 0.0098 and 27.28% *p* = 0.0103, respectively). However, neither the MS200 nor MS300 groups improved DA content (Fig. 1e). These results suggest that Sb conserves higher DA levels when administered orally in the 100 mg/kg dose.

### Oral Administration of Sb Improves Motor Deficits Induced by the PD Model

As clinical features of PD, there are two types of symptoms, non-motor and motor features, the last being the most important associated with a low quality of life [6]. To assess the potential neuroprotective effects of Sb in the improvement of motor dysfunctional MPTP-induced PD mice, we subjected mice to four behavioral motor tests.

**Fig. 2:**
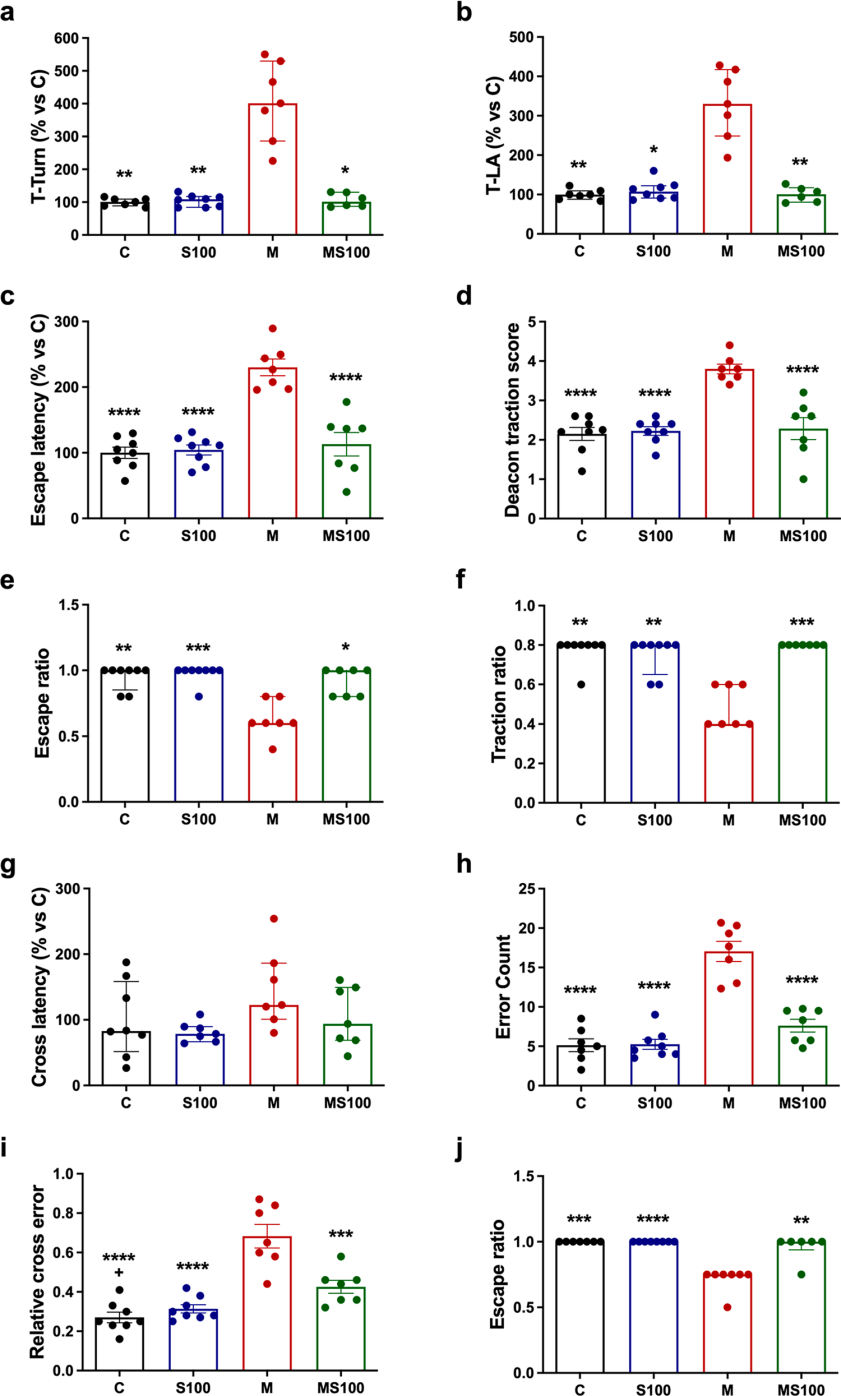
Sb improves motor behavior in MPTP-induced PD mice. A pole test was performed to evaluate bradykinesia and gross motor skills by measuring the time for turning down (T-turn; **a**, *n* = 6–8) and time for landing (T-LA; **b**, *n* = 6–8 ). A traction test was performed to evaluate balance, muscle strength, and fine motor skills by measuring escape latency (**c**, *n* = 7–8), Deacon’s scale (**d**, *n* = 7–8), escape ratio (**e**, *n* = 7–8), and traction ratio (**f**, *n* = 7–8). The beam test was performed to evaluate balance behavior and to confirm fine motor skills changes together with the traction test by measuring cross latency (**g**, *n* = 6–8), error count (**h**, *n* = 7–8), relative cross error (**i**, *n* = 7–8), and escape ratio (**j**, *n* = 7–8). Mice were treated with vehicles (C), silybin (S100), MPTP (M), or MPTP coadministered with silybin (MS100). Data (**a**, **b**, **e**, **f**, **g**, **j**) are presented as median with interquartile range and were analyzed by Kruskal-Wallis test with Dunn *post hoc* comparisons. Data (**c**, **d**, **h**, **i**) are presented as mean values ± SEM and were analyzed by One-way ANOVA with Tukey post hoc comparisons. **** *p* < 0.0001, *** *p* ≤ 0.001, and ** *p* ≤ 0.01, compared to M. # *p* ≤ 0.05 C compared to the MS100 group

The pole test was performed to measure the time descending the apparatus by taking two different parameters, T-Turn and T-LA, for evaluating bradykinesia and gross motor skills. For T-Turn, MPTP-intoxicated mice exhibited a locomotor deficit 305.55% slower than the control group (*p* = 0.0034), which significantly improved when co-administering Sb orally, reducing the time by 299.42% (*p* = 0.0270) (Fig. 2a). Identically, in T-LA, the M group exhibited a slow-down behavior of up to 229.40% compared to the control group (*p* = 0.0039), which improved with the Sb treatment gaining speed by 229.07% (*p* = 0.0063) (Fig. 2b). No significant differences were found between the control and S100 groups in both parameters, which suggests Sb has no improving effect on non-intoxicated mice. These results suggest that Sb treatment improves bradykinesia and associated gross motor skills in PD mice.

The traction test was performed to measure the mice’s ability to cross a metal rope to escape without falling to evaluate its equilibrium and muscle strength using different parameters. Rope escaping behavior was measured by two different but comparable parameters, the escape latency, which showed that MPTP mice take about 130.03% more time to escape from the middle of the rope compared to the control group (*p* = 0.0022) and 125.78% more time to escape compared to S100 (*p* = 0.0040), while Sb coadministration improved by 116.97% the escape latency (*p* = 0.0344) (Fig. 2c). The second parameter, Deacon’s traction score, showed that MPTP-induced PD mice have worst scores compared to the control (76.74 %, *p* = 0.0029) and S100 mice (73.25%, *p* = 0.0034) (Fig. 2d). Also, Sb coadministration improved motor dysfunction with a tendency to reduce its score by about 70.43% compared to M (*p* < 0.026) (Fig. 2d). These results demonstrate that Sb improved traction behavior and equilibrium measured by both parameters; furthermore, it demonstrated no differences between measuring the rope escape behavior by time latency or using the Deacon traction score. When measuring the dropout rate in the escape ratio, the M group presented considerable attrition behavior with about 33.83% less escape ratio compared to the control group (*p* = 0.0034), about 36.46% more attrition compared to S100 (*p* = 0.0008) (Fig. 2e). In comparison, Sb administration improved attrition behavior by 30.07% (*p* = 0.0302) (Fig. 2e). The C, S100, and MS100 groups had not statistical differences in all comparisons among them, which together suggests that Sb improves the dropout behavior in intoxicated mice and the escape ratio was a suitable parameter to determine it. The traction ratio was calculated to determine muscle strength. In this context, the MPTP-intoxicated group showed a higher fall index, translated into a lower traction ratio, about 37.33% lower compared to C (*p* = 0.0011), 34.10% lower compared to S100 (*p* = 0.0054) (Fig. 2f). Sb administration improved this traction ratio by 40.56% (*p* = 0.0003) (Fig. 2f), indicating that Sb administered orally can improve muscle strength in MPTP-intoxicated mice. C and S100 groups had no statistical differences in any of the parameters evaluated in the traction test, showing that Sb did not affect healthy mice behavior. In the traction test, data analysis showed that Sb administered orally improved equilibrium and muscle strength in MPTP-induced PD mice.

The third test, the beam test, measures the mice's ability to cross along a metal wire-wrapped bridge without failing or deserting, determining the time used for crossing (crossing latency), the number of errors by group, the relative cross error for improving test sensibility for detecting erratic behavior, and the escape ratio for detecting the dropout rate in experimental groups; all of them assessed fine motor skills and equilibrium behavior. Beam crossing behavior showed no significant differences between groups, but the MPTP group presented a higher latency (Fig. 2g). The error counting parameter showed a marked difference in MPTP-induced mice that increased miswalking by 266.12% compared to the control group (*p* < 0.0001) and 253.37% to S100 (*p* < 0.0001) (Fig. 2h). In contrast, Sb treatment improved walking behavior by 202.49% (*p* < 0.0001) (Fig. 2h), suggesting that Sb decreased miswalking in MPTP-intoxicated mice. In order to avoid the error miscount bias associated with variable time testing per mouse, the relative cross error was calculated to confirm differences between groups as revealed by the error count. As expected, differences were confirmed as MPTP exposure raised miswalking by 154.98% compared to the control group (*p* < 0.0001) and 139.07% compared to S100 (*p* < 0.0001), while improved with Sb coadministration by 96.49% (*p* = 0.0003) (Fig. 2i). This observation corroborated that Sb can improve miswalking and confirmed that the relative cross error could be a better parameter to measure due to the standardization of error count per second tested. Finally, regarding the attrition rate in the traction test, the M group had a more prominent dropout behavior with a significant 25% less escape ratio compared to both the control group (*p* < 0.0001) and S100 (*p* = 0.0002) (Fig. 2j). The Sb administration attenuates attrition completely in MPTP-intoxicated mice (*p* = 0.0063) (Fig. 2j). In addition, there were no significant differences between control, S100, and MS100 groups, which together suggests that oral Sb improves the dropout behavior only in intoxicated mice and had no effects in healthy mice.

Complementary beam test and traction test data showed that Sb alleviates motor dysfunction associated with miswalking, attrition, falling, and freezing, confirming fine motor skills improvement. Also, the traction test revealed equilibrium and muscle strength improvement by Sb oral administration in intoxicated mice.

In summary, these behavioral data indicate that Sb treatment appears to favor improvements of motor dysfunction in MPTP-induced PD mice by improving bradykinesia and associated gross and fine motor skills, maintaining balance and equilibrium behavior, and helping to preserve mice muscle strength.

### Sb Restores Mitochondrial Reductive Capacity and Decreases Lipid Peroxidation Caused by MPTP in Mice

Mitochondrial efficiency and viability are fundamental characteristics in the neuronal bioenergetic system associated with synaptic and brain functions [43, 44]. Compromised bioenergetics and, consequently, higher concentrations of mitochondrial reactive oxygen species (ROS) are some of the most common and earliest pathologic events in PD [45, 46].

**Fig. 3:**
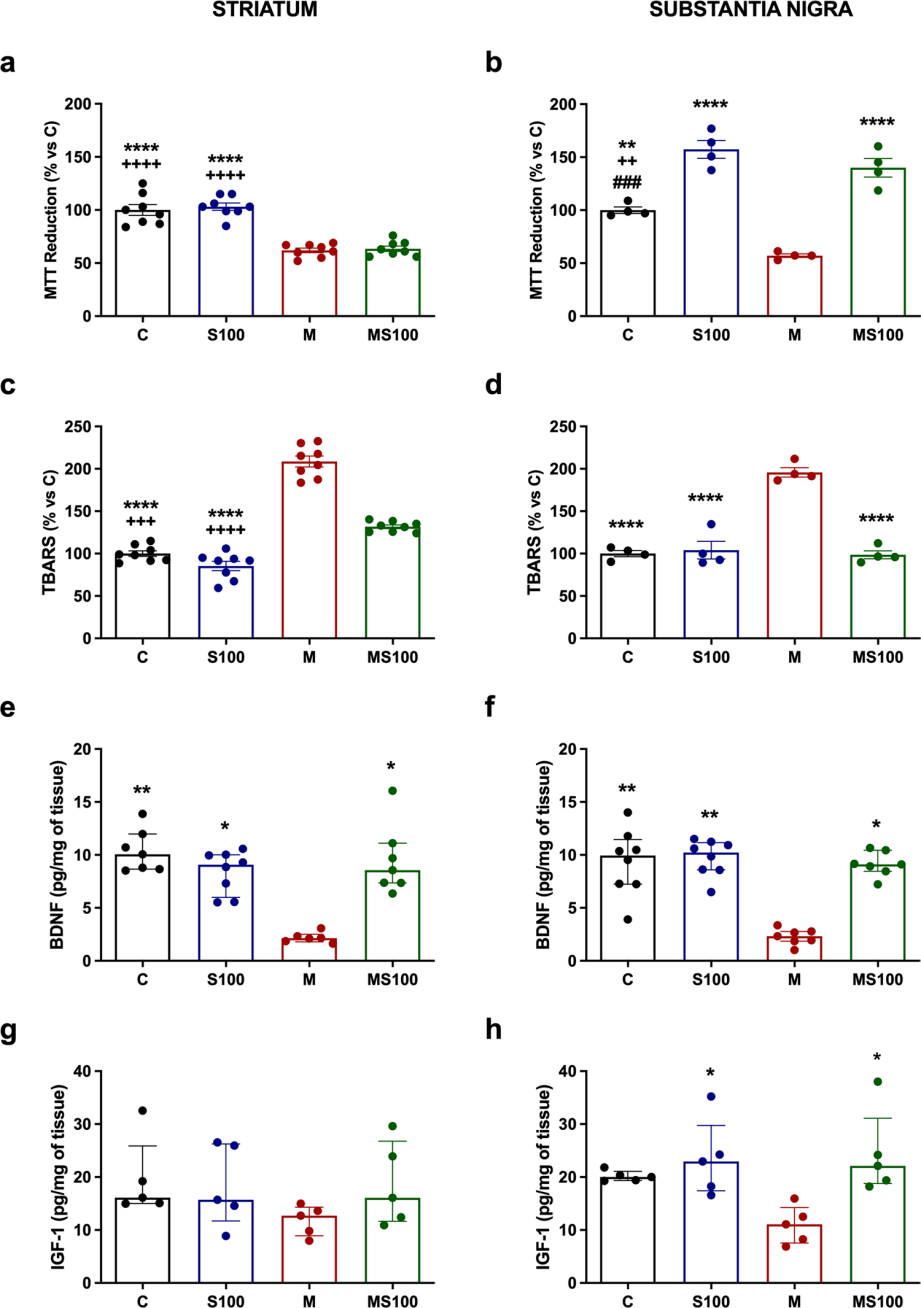
Sb treatment improves mitochondrial function, attenuates lipid peroxidation, and preserves neurotrophic factor levels in striatum and substantia nigra. Analysis of mitochondrial reduction power and availability in brain tissue by MTT Assay was performed in PD mice. Relative MTT-reduction percentages compared to the control group in the striatum (**a**, *n* = 8) and substantia nigra (**b**, *n* = 4) are shown. Quantification of malondialdehyde as a final lipid peroxidation product by the TBARS assay was also measured. Relative TBARS percentages compared to the control group in the striatum (**c**, *n* = 8) and substantia nigra (**d**, *n* = 4) were obtained. Neurotrophic factor levels were measured by ELISA from both neuronal areas. BDNF content in the striatum (**e**, *n* = 6–8) and substantia nigra (**f**, *n* = 7–8), and IGF-1 levels in the striatum (**g**, *n* = 5) and substantia nigra (**h**, *n* = 5) are presented. Mice were treated with vehicles (C), silybin (S100), MPTP (M), or MPTP coadministered with silybin (MS100). Data (**a**, **b**, **c**, **d**) are presented as means ± SEM and were analyzed by one-way ANOVA with Tukey post hoc comparisons. Data (**e**, **f**, **g**, **h**) are presented as median with interquartile range and were analyzed by Kruskal-Wallis test with Dunn post hoc comparisons. **** *p* < 0.0001, *** *p* ≤ 0.001, ** *p* ≤ 0.01, and * *p* ≤ 0.05 compared to the M group. ++++ *p* <0.0001, +++ *p* ≤ 0.001, and ++ *p* ≤ 0.01 compared to the MS100 group. ### *p* ≤ 0.001 compared to the S100 group

To examine Sb’s impact on the retention of mitochondrial availability and attenuation of reactive oxygen species, we determined mitochondrial reductive capacity (Fig. 3a, b) and TBARS (Fig. 3c, d) in the striatum and substantia nigra. In the case of the MTT reduction assay, both neuronal areas had a significant decrease when exposed to MPTP by 38.09% (*p* < 0.0001) and 42.89% (*p* = 0.0021) in the striatum and substantia nigra, respectively (Fig. 3a, b). In contrast, only Sb treatment increased the MTT reductive capacity by 82.92% in the substantia nigra (*p* < 0.0001), though no effect was observed in the striatum (Fig. 3a, b). This information suggests that Sb can act in substantia nigra by restoring or preserving the mitochondrial function measured by MTT reduction, whereas the striatum had no significant changes, maybe due to less mitochondrial density caused by synapses lost in this region [47]. Sb also enhanced mitochondrial function statistically different from the control group in both intoxicated mice by 40.03% (*p* = 0.0036) and healthy mice by 57.35% (*p* = 0.0002), indicating that Sb can promote better reductive efficiency in healthy and diseased mitochondria (Fig. 3b).

TBARS assay was measured in both striatum (Fig. 3c) and substantia nigra (Fig. 3d) to evaluate Sb’s impact on lipid peroxidation secondary to ROS formation. As expected, MPTP administration caused an increase in the amount of MDA by 108.65% in the striatum (*p* < 0.0001) and 95.82% in the substantia nigra (*p* < 0.0001) (Fig. 3c, d) as a typical response of brain tissue to oxidative [12, 48]. Contrasting to MTT assay results, TBARS decreased in both neuronal tissues with Sb treatment, about 76.94% in the striatum (*p* < 0.0001) and 97.26% in substantia nigra (*p* < 0.0001) (Fig. 3c, d). Sb attenuated MDA levels secondary to lipid peroxidation measured by TBARS assay. Altogether, Sb can act directly in both the striatum and substantia nigra as an antioxidant, improving mitochondrial function only in the substantia nigra, while reducing oxidative species produced by neuronal stress in both regions.

### Sb Preserves BDNF and IGF-1 Levels in the Striatum and Substantia Nigra Affected by MPTP Exposure in Mice

Neurotrophic factors are proteins that support neuronal plasticity, development, maturation, and survival [49]. Studies from animal PD models showed that neurotrophic factors such as BDNF, GDNF, and IGF-1, among others, can protect dopaminergic neurons from neurodegeneration promoted by MPTP and 6-OHDA [50]. Furthermore, clinical studies reported reductions in neurotrophic factor levels and associated them with the severity of motor and non-motor symptoms. Neurotrophic factor decline is crucial in PD progression and can be a potential therapeutic target [51]

To evaluate the effect of Sb on the levels of neurotrophic factors, we measured BDNF (Fig. 3e, f) and IGF-1 (Fig. 3g, h) in both the striatum and substantia nigra from healthy and MPTP-exposed mice. BDNF levels were significantly decreased in intoxicated mice compared to controls (Fig. 3e, f); a reduction of about 78.87% in the striatum (*p* = 0.0013) and 75.56% in substantia nigra (*p* = 0.0054) was detected (Fig. 3e, f). Sb co-administration restored basal BDNF levels in both nuclei, striatum (*p* = 0.0211), and substantia nigra (*p* = 0.0285), confirming that Sb preserves basal BDNF amount. No significant differences were found between C and S100 groups in all experiments, suggesting that Sb did not improve the effect in non-intoxicated mice. On the other hand, Sb administration increased IGF-1 levels in substantia nigra from healthy mice by 16.22% (*p* = 0.0327) and MPTP-treated mice by 20.85% (*p* = 0.0234), compared to PD mice (Fig. 3h). These results suggest that Sb can re-establish both neurotrophic factors, mainly BDNF, that were affected by the MPTP treatment in mice.

### Sb Attenuates Neuroinflammation by Diminishing TNFα, IL-1β, and IL-6 Levels Without Modifying IL-10 Content and Enhances IL-4 and Fractalkine Production in PD Mice

**Fig. 4:**
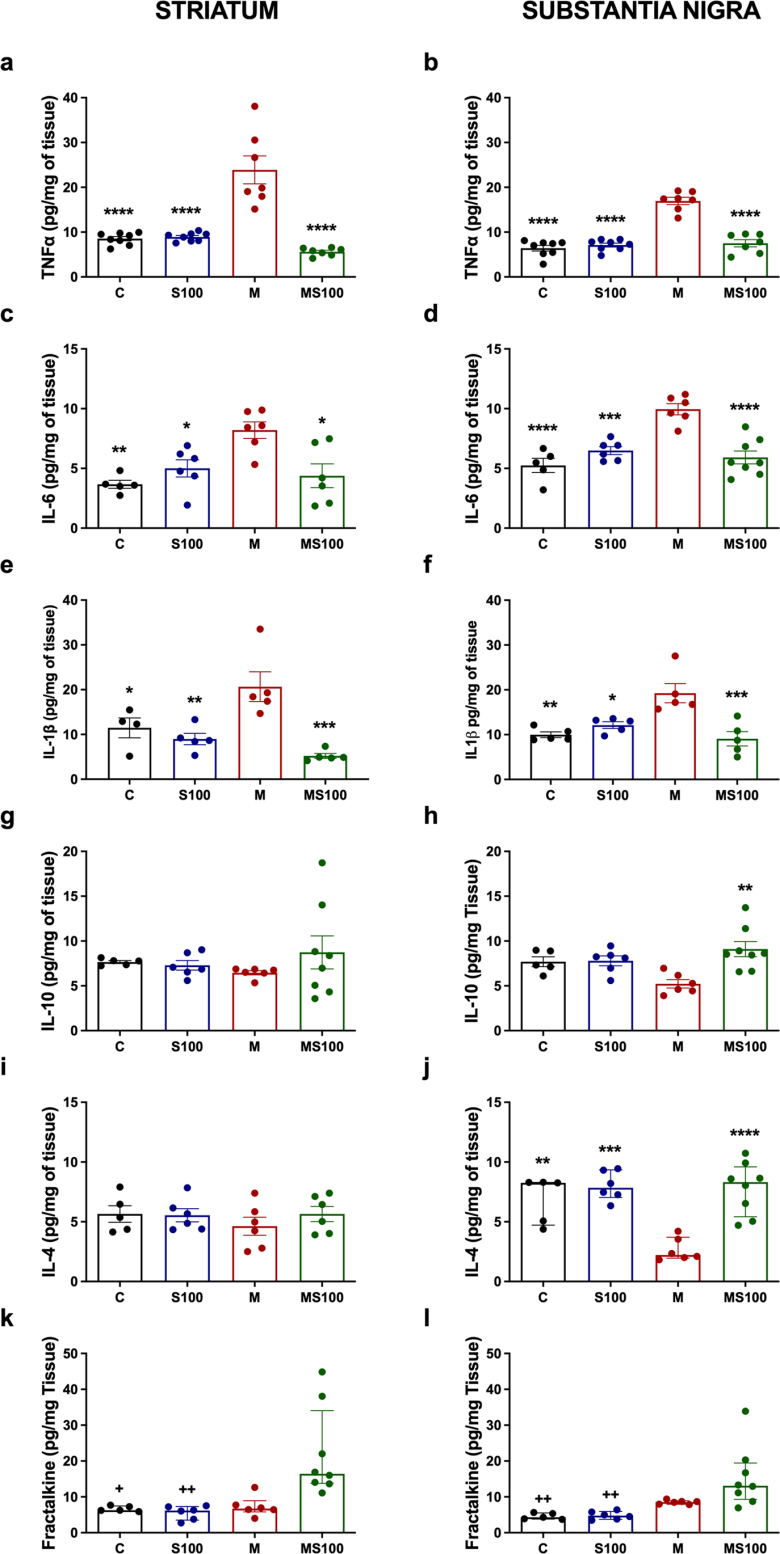
Sb treatment reduces MPTP-induced neuroinflammation by TNFα, IL-1β, and IL-6 and enhances IL-4 and fractalkine levels in MPTP-induced PD mice. Cytokine profiles were measured in the striatum (**a**, **c**, **e**, **g**, **i**, **k**) and substantia nigra (**b**, **d**, **f**, **h**, **j**, **l**) of PD mice. TNFα (**a**, *n* = 7–8; **b**, *n* = 7–8), IL-6 (**c**, *n* = 6; **d**, n = 6), IL-1β (**e**, *n* = 5; **f**, n = 5), IL-10 (**g**, *n* = 6; **h**, *n* = 6), IL-4 (**i**, *n* = 6; **j**, *n* = 6), and fractalkine (**k**, *n* = 6; **l**, *n* = 6) were determined in homogenized brain tissues by capture ELISA. Mice were treated with vehicles (C), silybin (S100), MPTP (M), or MPTP co-administered with silybin (MS100). Data (**a**, **b**, **c**, **d**, **e**, **f**, **g**, **h, i**, **j**) are presented as means ± SEM and were analyzed by one-way ANOVA with Tukey post hoc comparisons. Data (**k**, **l**) are presented as median with interquartile range and were analyzed by Kruskal-Wallis test with Dunn post hoc comparisons. **** *p* < 0.0001, *** *p* ≤ 0.001, ** *p* ≤ 0.01, and * *p* ≤ 0.05 compared to the M group. ++ *p* ≤ 0.01, and + *p* ≤ 0.05 compared to the MS100 group

Neuroinflammation is another of the most common and relevant processes in neurodegenerative disorders, especially in PD pathogenesis [52–54]. To investigate whether Sb modulates the increased proinflammatory cytokines in PD, enhancing anti-inflammatory cytokine levels, we examined TNFα, IL-6, IL-1β, and anti-inflammatory IL-10, IL-4, and fractalkine levels in both nuclei, striatum, and substantia nigra. MPTP-intoxicated increased the TNFα levels in the striatum by 181.84% (*p* < 0.0001) and in the substantia nigra by 156.97% (*p* < 0.0001) (Fig. 4a, b); IL-6 in the striatum by 223.50% and in substantia nigra by 89.60% (*p* = 0.0299 and *p* < 0.0001, respectively) (Fig. 4c, d); and IL-1β in the striatum by 121.21% and in the substantia nigra by 93.01% (*p* = 0.0409 and *p* = 0.0015, respectively) (Fig. 4e, f), compared to the control group. These observations confirmed a substantial proinflammatory microenvironment in the MPTP-treated mice. The Sb coadministration re-established basal levels of all proinflammatory cytokines TNFα (striatum, *p* < 0.0001; substantia nigra *p* < 0.0001) (Fig. 4a, b), IL-6 (striatum, *p* = 0.0085; substantia nigra *p* < 0.0001) (Fig. 4c, d), and IL-1β (striatum, *p* = 0.0004; substantia nigra *p* = 0.0006) (Fig. 4e, f). These data suggest that Sb attenuates neuroinflammation in MPTP-intoxicated mice. On the other hand, anti-inflammatory IL-10 and IL-4 levels showed no change in MPTP mice in the striatum (Fig. 4g, i). Also, IL-10 was not significantly reduced in the substantia nigra of MPTP-exposed mice (Fig. 4h), whereas Sb significantly raised IL-10 in the substantia nigra of MPTP mice (*p* = 0.0022) (Fig. 4h). Nonetheless, in substantia nigra, the MPTP administration reduced IL-4 levels by 61.05% (*p* = 0.0029), which were re-established to basal level when Sb was co-administered (*p* < 0.0001) (Fig. 4j). In the case of fractalkine, there were no modifications when mice were exposed to MPTP in both the striatum and substantia nigra (Fig. 4k, l). In contrast, Sb administration enhanced fractalkine levels in the striatum (*p* = 0.0191 compared to the control group, *p* = 0.0018 compared to the MS100 group) and substantia nigra (*p* = 0.0022 compared to the control group, *p* = 0.002 compared to the MS100 group) of MPTP mice (Fig. 4k, l).

These data indicate that Sb treatment attenuates neuroinflammation directed by TNFα, IL-1β, and IL-6 by promoting IL-10 and IL-4 only in the substantia nigra, and fractalkine in both brain structures [53, 55].

## Discussion

Silymarin and Sb have been widely described as potential antioxidants and anti-inflammatory therapies in many diseases, including neurodegenerative diseases [18, 20, 56–58]. The present study demonstrated the neuroprotective properties of Sb in the MPTP-induced mice PD model. Our results show that Sb prevented DA depletion in the striatum when orally administering 100, 400, and 500 mg/kg, presenting higher effects at 100 mg/kg, preserving more than 50% of DA (Fig. 1) content, which corresponds to the results obtained by Pérez-H and colleagues in 2014, who found that silymarin administered intraperitoneally in the MPTP mice model retained about 60% of DA content [15]. This information complements our study by assuming that most of the neuroprotective effects of silymarin are mediated by Sb, considering the dose loss caused by intestinal absorption and first-step metabolism [58, 59]. A “J-shaped” biphasic dose-response relationship was observed, suggesting that an increase in the Sb amount between 200 and 400 mg/kg causes loss of its neuroprotective effects with less neuroprotection in doses higher than 400 mg/kg, maybe due to oxidative-mediated hormesis [60–62], suggesting biological mechanisms based on different molecular affinities and activation for Sb, as shown on other biological systems such as receptors for estrogen, prostaglandin, and TNFα [63]. Our results of DA restoration by Sb oral administration correspond with the dopaminergic neuron rescue effect documented before with orally administered silybin in another study [21].

Neurotrophic factors such as BDNF and IGF-1 play a critical role in the development, proliferation, insulin-like signaling, pro-survival, antioxidant, and neuroprotection of dopaminergic neurons [64, 65]. In addition, in vitro, and in vivo PD models showed that both BDNF and IGF-1 promoted the survival of dopamine neurons and improved motor symptoms [66–69]. In this study, Sb administration also restored IGF-1 and BDNF content diminished by MPTP administration (Fig. 3). These findings correlated with improved gross motor skills, muscle strength, fine motor skills, and balance behavior in the MPTP-intoxicated mice (Fig. 2), suggesting a significant symptomatic improving effect exerted by Sb, restoring not only DA content but also increasing pro-survival factors.

Mitochondrial injury and REDOX balance dysfunction are essential parts of PD pathogenesis, described as the oxidative stress hypothesis, which consists of high levels of ROS associated with dopaminergic neuronal mitochondria systems promoting neuronal death by enhanced biomolecule damage [5, 20]. Neuronal death, damage signals, and degenerative microenvironment activate astrocytes and microglia, promoting enhanced neuroinflammation in early stages and chronic low-neuroinflammation in late PD progression stages [4, 54]. Some hypotheses suggested that neurodegeneration in PD could be prevented or attenuated by the production and release of neurotrophic factors, accompanied by inhibiting neuroinflammation and oxidative stress mediators [55]. Our results showed that the MPTP model decreased the mitochondrial reductive capacity and cell viability (Fig. 3). In contrast, Sb improved it in substantia nigra, probably acting as an antioxidant agent by diminishing pro-oxidant biomolecules—mainly lipids—detected in both the striatum and substantia nigra, as a lower amount of MDA [70]. These results agree with previous studies, where Sb acted as a potent antioxidant in in vivo PD models [20, 21, 71] and AD models [72]. In addition to oxidative damage prevention, Sb also decreased the proinflammatory microenvironment developed by MPTP (Fig. 4) by increasing anti-inflammatory IL-10 and IL-4 content, thus indicating that Sb could act as an anti-inflammatory agent by inhibiting TNFα, IL-1β, and IL-6 synthesis which activate pathways like the NF-kB/IRE-IKBɑ pathway. In addition, Sb can antagonize the proinflammatory cytokines signaling, such as upregulation of microglial regulation mechanisms as fractalkine, as shown in our study, where fractalkine ligand levels were increased only in MPTP-induced PD mice. These results agree with previous findings on the Sb role in PD by attenuating inflammasome, autophagy, and oxidative stress [21] while enhancing IL-10 and IL-4 production in an AD model [73]. Further studies are needed to identify Sb’s impact on microglia-dependent neuroinflammation development and control mechanisms exerted by astrocytes, considered the primary neurotrophic factors providers, which enhance pro-survival mechanisms when neuroinflammation or neurotoxic stimuli occur [74]. Although Sb neuroprotective mechanisms remain unknown, there is information about Sb activity that can explain how Sb ameliorates PD biochemical and motor outcomes. Sb is a direct free radical-scavenger agent by its reduction to 2,3-dehydrosilybin; this could be a direct mechanism as a potential antioxidant and explain how Sb can reduce the amount of lipid peroxidation products and diminish oxidative stress [75]. In addition, some studies identified Sb as an agonist of the estrogen beta receptor and how it can be an immunomodulator agent through estrogen pathways [76, 77]. Further, Sb can directly inhibit STAT3 inflammatory pathway [78]. Also, silymarin is widely described in different neurological and non-neurological related diseases as an activator and enhancer of the Nrf2 pathway and inhibitor of the NF-kB, NLRP3, and JNK pro-inflammatory pathways [75–79]. In addition, although isolated Sb activity needs to be further evaluated, Sb can be the primary molecule that conserves these bioactive properties [as thoroughly reviewed in 79]. In this context, Sb can act as an antioxidant and anti-inflammatory agent by regulating these pathways and increasing neurotrophic factor contents, translating into preserving DA levels and improving motor behavior.

PD is a multifactorial disease in which many energetic, inflammatory, and homeostatic systems are impaired. Natural product-derived molecules are interesting for therapeutic approaches due to their multi-target activities, then modulating different outcomes simultaneously [80]. Popular molecules, such as caffeine, quercetin, baicalein, ginseng, and curcumin, have been evaluated as potential neuroprotectors and have demonstrated beneficial effects in vivo e in vitro [81]. However, their effects are mainly related to exogen and endogen antioxidant properties and subsequently diminished neuroinflammation due to reduced oxidative stress damage [82]. In contrast, Sb has therapeutical activities through different pathways in addition to the classical antioxidant Nrf2 and anti-inflammatory NF-kB pathways studied in other diseases. Sb can be proposed as a modulator of PD progression through different mechanisms of action, such as the estrogenic or the neurotrophic signals and pathways for neuroprotection.

**Fig. 5:**
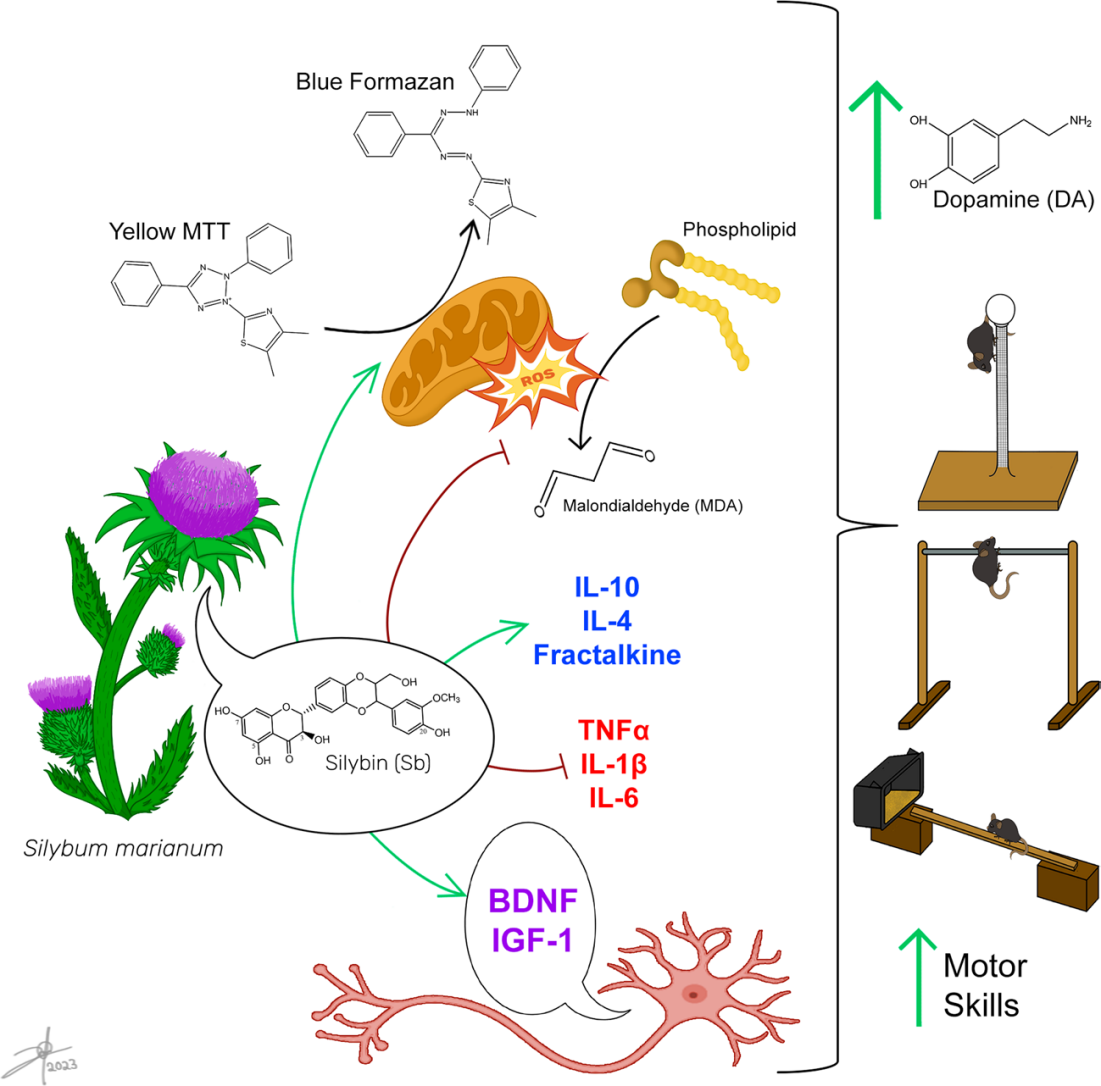
Oral administered silybin alleviates PD motor and biochemical outcomes in the MPTP-induced PD mice model. Sb preserves striatal dopamine content and improves bradykinesia, fine and gross motor skills, muscle strength, balance behavior, and motor coordination, acting as a neuroprotective agent by enhancing mitochondrial reduction power, neutralizing oxidative stress detected by MDA levels, diminishing neuroinflammation directed by TNFα, IL-1β, and IL-6, enhancing IL-10, IL-4 and fractalkine responses and preserving BDNF, and IGF-1 content in PD model mice

In conclusion, our findings demonstrate the bioactive effects of Sb, which confirmed it as a potential neuroprotective molecule in an MPTP-induced model of PD in mice (Fig. 5). Sb’s most effective oral dose was 100 mg/kg, which preserved DA on striatal neurons, improving motor deficits as gross and fine motor skills, equilibrium, and muscle strength, also restoring IGF-1 and BDNF content. The mechanism of Sb neuroprotection was described in this work as a preserver of mitochondrial function, attenuating lipid peroxidation and diminishing neuroinflammation caused in both the striatum and substantia nigra. Therefore, based on our results and the information previously reported in the literature, we propose that Sb administered orally may be potentially a pharmacological alternative for PD treatment.

## Data Availability

The datasets used and/or analyzed during the current study are available from the corresponding author on reasonable request.
